# Foetal haemoglobin and disease severity in sickle cell anaemia patients in Kampala, Uganda

**DOI:** 10.1186/1471-2326-12-11

**Published:** 2012-09-07

**Authors:** Lena Mpalampa, Christopher M Ndugwa, Henry Ddungu, Richard Idro

**Affiliations:** 1 Life Link Medical Centre, Kampala, Uganda; 2 Department of Paediatrics, Makerere University, College of Health Sciences, Kampala, Uganda; 3 Department of Medicine and Uganda Cancer Institute, Mulago Hospital, Kampala, Uganda; 4 Department of Paediatrics, Mulago Hospital/ Makerere University, College of Health Sciences, Kampala, Uganda; 5 Centre for Tropical Medicine, Nuffield Department of Medicine, Oxford University, Oxford, UK

**Keywords:** Sickle cell anaemia (SCA), Foetal haemoglobin (HbF), Disease severity

## Abstract

**Background:**

Sickle cell anaemia (SCA) is a major chronic health problem in Uganda. In patients with SCA, the level of foetal haemoglobin (HbF) has been found to be important in influencing the clinical course of the disease. Thus populations with high levels of HbF like those in Saudi Arabia have been described as having a milder clinical course with fewer complications as compared to populations with lower levels. Disease modifying drugs can increase the Hb F levels and modify the presentation of SCA.

**Methods:**

This was a cross sectional study in which we determined foetal haemoglobin levels and examined the relationship between HbF levels and disease severity in SCA patients in Mulago Hospital, Kampala, Uganda. We consecutively enrolled 216 children aged 1 year to 18 years with SCA attending the Sickle Cell Clinic at Mulago Hospital whose guardians had given consent. The history included age at onset of initial symptoms and diagnosis, number of hospitalisations and blood transfusions and other complications of SCA (cardiovascular accidents, avascular hip necrosis and priapism). A detailed physical examination was performed to assess the current state and help describe the disease severity for each patient. Blood samples were drawn for HbF levels. HbF levels ≥10% was defined as high.

**Results:**

Of the 216 children, (80) 37% had HbF levels ≥10%. Significant correlations were observed between HbF level and several clinical parameters independent of age including age at diagnosis (p value 0.013), number of hospitalisations (p value 0.024) and transfusions (p value 0.018) since birth.

**Conclusion:**

A third of the children with SCA attending the Sickle cell clinic in Mulago Hospital have high HbF levels. Higher HbF level is associated with later onset of symptoms and presentation, and less severe disease characterised by fewer hospitalisations and blood transfusions. We suggest HbF levels should be determined at initial contact for patients with SCA to guide counselling and identify those who may need closer follow up and consideration for disease modifying drugs.

## Background

Sickle cell anaemia (SCA), the most common inheritable disease in Africa, is a leading public health problem in the region and elsewhere where descendants of Africans have settled [1]. Worldwide, it is recognized as a major cause of morbidity and mortality with tremendous social and economic impact mainly due to the recurrent acute episodic clinical events called “crises” and hospitalizations [2]. In Africa, SCA is estimated to contribute to an equivalent of 5% of under-five deaths, with up to 16% in some countries such as Nigeria and only half of the affected children live beyond their fifth birthday [3].

In Uganda, SCA is the most frequent hereditary haemolytic anaemia. Unlike the situation in developed countries, where there has been a reduction in morbidity and mortality mainly because of the availability of specialised comprehensive care in these countries [4], in Uganda there is no neonatal screening program and care is mainly supportive. The disease is therefore associated with high morbidity and mortality with about 70 – 80% of children with the disease dying before the age of two years [5]. Several studies in the 1950s estimated that 20% of the population had the sickle cell trait [6]. The gene frequency had marked variation between different tribal groups, being highest among the Bamba (40%), 16-20% among the Baganda, Itesot, Acholi and lowest among the Karamajong (1-4%) [6,7].

One important factor that has been described as influencing the clinical course and hence disease severity in SCA is foetal haemoglobin (HbF) level [8]. Populations with high HbF levels like those in Eastern Saudi Arabia have been described as having less severe disease with fewer complications [9] and better survival [10]. Foetal haemoglobin interferes with the polymerization of HbS which usually occurs at low oxygen tension in patients with SCA [11]. In addition, red blood cells with higher HbF content have prolonged survival hence patients with high Hb F level also tend to have higher overall Hb levels [12].

As part of comprehensive care, disease-modifying drugs (DMDs), most of which act by inducing and raising HbF levels, are now being used to influence the clinical picture and quality of life in patients with SCA. These drugs have been shown to be beneficial especially in those with severe disease [11].

The level of HbF is fairly characteristic of the child’s level through life after the first year of life and hence this value has been postulated to be of prognostic importance and predict survival [10,13]. Although the baseline HbF levels are known in several populations around the world, hardly any studies have examined HbF levels in patients in our region or how this may correlate with clinical disease severity. Such knowledge would be quite important in assessing the possible impact that introducing DMDs may have.

In this study, therefore, we aimed to determine the HbF levels in Ugandan children with SCA and examine the relationship between HbF level and disease severity.

## Methods

### Design

This was a cross sectional study that examined the relationship between HbF level and disease severity.

### Study setting

The study was conducted at the Sickle Cell Clinic (SCC) of Mulago Hospital over a two month period from January to February 2010. Located in the capital, Kampala, Mulago is the National Referral Hospital for Uganda and teaching hospital for Makerere University College of Health Sciences The hospital has a bed capacity of 1,500 and an annual inpatient turnover of 120,000. The SCC is the only such clinic in Uganda which provides specialized care for both children and adults with SCA. The clinic runs cost-free daily services and receives about 250–300 patients each week. It has over 6000 registered patients. Patients are registered after confirmation of homozygous SS disease by Hb electrophoresis on cellulose acetate membranes.

The clinic functions as a regular follow up centre and a day care centre, where non-critically ill patients are admitted for daytime management. Very ill patients are admitted to the paediatric emergency ward (Acute Care Unit) or adult medical casualty unit. The services provided include counselling, blood transfusions, and provision of medicines e.g. antimalarial prophylaxis, folic acid and analgesics. The clinic relies on volunteers for the delivery of some of the services. Disease modifying drugs such as hydroxyurea are not routinely provided for eligible patients.

### Study participants

All children aged between 1 and 18 years with confirmed SCA (Hb SS) attending the clinic during the study period were eligible. The age of recruitment was from one year because after the first year, the HbF level is fairly characteristic for a patient and essentially remains so throughout life [13]. All children whose parents gave a written informed consent were included in the study. We excluded from the study any child who was severely anaemic (Hb <5 g/dl). Patients who met the study criteria were consecutively enrolled daily. A sample size of 211 was calculated using Kish Leslie formula for cross sectional surveys using 17% as the expected prevalence of high HbF level in the cohort [14]. High HbF was defined as foetal haemoglobin ≥ 10% as used in earlier studies in Nigeria [14], Senegal [15] and Saudi Arabia [16].

### Study procedure

Identification and screening of eligible patients was carried out by a study nurse at the reception. Parents or guardians of patients who met the selection criteria were then approached for consent by the study nurse and, in addition, assent was sought from children 8 years or older. Patients whose parents/guardians gave consent were then recruited and assigned a study number. Data from clinical history (from both parents/guardians and available in the clinical notes) and physical exam was abstracted on to a pre-designed standard case record form. The descriptions included age at onset of symptoms, number of transfusions, hospitalisations, and severe pain episodes in the past year; complications such as cerebrovascular accidents, priapism, and avascular necrosis; and findings on physical examination.

Four millilitres of peripheral venous blood was drawn for a complete blood count and foetal haemoglobin levels. The blood was put in two ethylenediaminetetraacetic acid (EDTA) bottles; one for an automated complete blood count and the second for determination of Hb F levels by the alkali denaturation method by Betke [17]. This test has been used in other studies of HbF levels in populations in Nigeria by Fatunde et al [14], and in Senegal by Diop et al [15]. The basis of this test is that foetal haemoglobin (HbF) is more resistant to denaturation in alkaline solution than adult haemoglobin (HbA). Alkali converts HbA to alkaline hematin. Alkaline hematin is insoluble and precipitates. HbF is quantitated by measuring the haemoglobin concentration before and after denaturation. The procedure involves treating a cyanmethaemoglobin solution with 1.2 N sodium hydroxide. Saturated ammonium sulphate is used to neutralise the alkali after 2 minutes and the mixture is filtered, read at 420 nm and compared with an adequate dilution of the original cyanmethaemoglobin solution in water. All samples were analysed on the collection day.

### Data analysis

All case record forms were checked for completeness and accuracy before each participant left the clinic. The corrected forms were then kept in a study file. The data recorded on each form was then entered into a database using EPI-data version 3.1 package using double data entry to ensure correctness. Data was exported to and analysed using STATA version 10 software (STATA Coop, TX). Proportions were compared using Pearson’s chi square test and reported with the odds ratios with 95% confidence intervals.

Normally distributed continuous variables were summarized as means and compared using the student’s t test while the median was used for skewed data and compared using the Mann Whitney U / Rank sum test. In all cases, p values <0.05 was considered significant.

### Ethical considerations

Approval to conduct the study was obtained from Makerere University School of Medicine Research and Ethics Committee. Written informed consent was obtained from legal caretakers of participants and assent was sought from participants aged 8 years and above before enrolment into the study. Enrolment was voluntary and participants could withdraw from the study at any time without consequences to the patient. Results of the investigations and any additional important information were availed to the primary physicians taking care of the study participants.

## Results and discussion

### Results

Over a two month period, from January to February 2010, five hundred and sixty eight patients attended the Sickle cell clinic in Mulago Hospital and 216 met our eligibility criteria and were enrolled. Of the 216 study subjects, 115 (53.2%) were female and 101 (46.8%) male. The mean (SD) age was 9.3 (4.8) years and the median age 9 (IQR 5–13) years. The characteristics of the study participants are summarised in Table 1

**Table 1:** Clinical characteristics of SCA patients by HbF level

**Characteristic**	**Patients with High HbF levels (≥10%) N = 80(%)**	**Patients with low HbF levels (<10%) N = 136 (%)**	**OR (95% CI)**	**p value**
**Demographic features**				
**Gender**				
Female	48(60%)	67(49.3%)	1.54 (0.88-2.71)	0.128
Male	32(40%)	69(50.7%)		
**Age**				
<5 years	28 (35%)	20 (14.7%)	3.12 (1.58 -6.17)	**0.0005**
≥ 5 years	52 (65%)	116 (85.3%)		
HISTORY				
**Reason for clinic visit**				
Sick visit	63(77%)	100 (73.5%)	1.33 (0.69-2.57)	0.390
Routine visit	17 (23%)	36 (26.5%)		
**Duration of follow up in SCC**				
< 5 years	54 (67.5%)	52 (38.2%)	3.36 (1.83-6.16)	**<0.001**
≥ 5 year	26 (32.5%)	84 (61.8%)		
**Transfusions since birth**				
<3	58 (72.5%)	83 (61%)	1.68 (0.91-3.08)	0.080
≥ 3	22(27.5%)	53 (39%)		
**Hospitalisations since birth**				
≤2	48 (60%)	61(55%)	1.82 (1.03- 3.21)	**0.036**
>2	32 (40%)	75 (45%)		
SIGNS				
**Jaundice**				
present	53(66.3%)	85 (62.5%)	1.18 (0.66-2.11)	0.580
absent	27 (33.7%)	51 (37.5%)		
**Palpable spleen**				
Yes	24 (30%)	28 (20.6%)	1.65 (0.87-3.13)	0.119
No	56 (60%)	108 (79.4%)		
**Hepatomegaly**				
Present	40 (50%)	60 (44.1%)	1.27 (0.73-2.21)	0.404
absent	40 (50%)	76 (55.9%)		
**Haematological parameters**				
**WBC count**				
<15.0	35 (43.8%)	65 (47.8%)	1.18 (0.67-2.05)	0.566
≥ 15.0	45 (56.2%)	71 (52.2%)		
**RBC count**				
<3.0	48 (60%)	80 (58.8%)	1.05 (0.60-1.85)	0.865
≥ 3.0	32 (40%)	56 (41.2%)		
**Hb level**				
≥7 g/dl	60 (75%)	98 (72.1%)	1.16 (0.62-2.19)	0.638
<7 g/dl	20 (25%)	38 (27.9%)		
**PLT level**				
<400	40 (50%)	66 (48.5%	1.06 (0.61-1.85)	0.835
≥400	40 (50%)	70 (51.5%)		

### Foetal haemoglobin levels

The mean HbF level of the participants was 9.0% (SD 5.58) and the median was 7.9% (IQR 4.7-12.4%). Eighty patients (37.0%) had high levels of Hb F (≥10%). The majority of the participants had HbF levels between 2 and 11% with very few having levels greater than 20%. Patients 5 years and younger on average had higher HbF levels [mean HbF 11.9% (SD 5.69)] compared to older patients [mean HbF 7.9% (SD 5.13)] although this difference was not statistically significant (p value 0.843).

Overall, high HbF level was associated with less severe disease. In the past year, children with high HbF levels had significantly fewer hospitalisations (p value 0.036) as well as fewer transfusions and painful episodes, but this relationship was not statistically significant (Table 2). Furthermore, patients with high HbF were less likely to have had dactylitis or stroke; and of the patients who developed dactylitis, patients with high HbF levels were more likely to develop this at an older age, but this relationship was not statistically significant. There were only 2 patients with avascular necrosis and both had low HbF levels and 3 with a history of priapism, of which 2 had low levels HbF levels.

**Table 2:** Features of disease severity by HbF level

**Clinical features**	**Patients with High HbF levels (≥10%) N = 80(%)**	**Patients with low HbF levels (<10%) N = 136 (%)**	**OR (95% CI)**	**p value**
**History of dactylitis**				
Present	53 (66.3%)	106 (77.9%)		
Absent	27 (33.7%)	30 (22.1%)	1.80 (0.97-3.53)	0.060
**Age at onset of dactylitis**				
<1 year	37 (69.8%)	78 (73.6%)		
≥ 1 year	16 (30.2%)	28 (26.4%)	1.20 (0.57-2.50)	0.617
**Age at diagnosis**				
≤ 1 year	31 (38.8%)	54 (40%)		
> 1 year	49 (61.2%)	81 (60%)	1.05 (0.59-1.86)	0.857
**Hospitalisations in past year**				
No	40 (50%)	63 (46.3%)	1.16 (0.67-2.02)	0.602
yes	40 (50%)	73 (53.7%)		
**Transfusions in past year**				
No	62 (77.5%)	92 (67.7%)	1.65 (0.87-3.13)	0.123
Yes	18 (22.5%)	44 (32.3%)		
**Painful episodes in past year**				
No	27 (33.7%)	41 (30.1%)	1.18 (0.65-2.13)	0.583
Yes	53 (66.3%)	95 (69.9%)		
**History of stroke**				
No	76 (95%)	123 (90.4%)	2.00 (0.63-6.43)	0.231
Yes	4 (5%)	13 (9.6%)		

However, when the relationship between HbF level as a continuous variable and some of the markers of disease severity was examined, a statistically significant negative correlation was observed between HbF level and participant age (r = −0.299, p value = <0.0001), the total number of transfusions (r = −0.181, p value 0.004), all cause hospitalisations (r = −0.173, p value 0.006), and number of severe pain episodes in the past year (r = −0.104, p value 0.020). The association between HbF and age at diagnosis (r = 0.151, p value 0.013), all cause hospitalisations (r = −0.135, p value 0.024) and need for transfusions (r = − 0.144, p value 0.018) since birth remained significant even when this was adjusted for age of the participants Table 3.

**Table 3:** Correlation between some of the cinical characteristics and HbF levels

***Variable***	**HbF Levels**	***p-value***	**Adjusted for age**	
	***Pearson Coefficient***		***Pearson Coefficient***	***p-value***
Age	***−0.299***	***0.000***	***-***	***-***
Age at diagnosis of SCA	0.047	0.245	***0.151***	***0.013***
Age at onset of dactylitis	−0.038	0.291	0.109	0.086
Number of hospitalisations since diagnosis	***−0.173***	***0.006***	***−0.135***	***0.024***
Number of transfusions since diagnosis	***−0.181***	***0.004***	***−0.144***	***0.018***
Number of intra-muscular injections received in past year	***−0.140***	***0.020***	−0.099	0.075
Number of hospitalizations in the past year	−0.068	0.161	−0.104	0.064
Number of blood transfusions in the past year	−0.038	0.291	−0.071	0.149
WBC Count	0.074	0.138	−0.023	0.367
Hb levels	−0.003	0.480	0.024	0.364
PLT levels	−0.105	0.061	−0.105	0.062

### Discussion

The influence of foetal haemoglobin levels on disease manifestation in SCA has hardly been studied in East Africa. The objective of our study was to examine the relationship between HbF level and disease severity among patients with SCA attending the Sickle cell clinic in Mulago Hospital, Uganda. We found that over one third of the patients studied had high levels of Hb F and that higher levels were associated with less severe disease. In particular, higher levels were associated with older age at diagnosis, fewer all cause hospitalisations, transfusions and severe pain episodes.

The proportion of patients with high HbF levels, 37%, was higher than what was observed in West Africa, particularly Senegal (25%) [15] and Nigeria (17%) [14]. This is surprising because patients from Senegal are thought to have the Senegal haplotype, which is thought to be associated with a higher HbF level. One difference is that the Dakar study had adult participants and this may account for the slight difference in Hb F levels, even though Hb F levels are thought to be fairly stable after the first year of life.

The average HbF level in our population was 9.0% which is comparable to other studies in Congo [18], Nigeria [19], and Saudi Arabia [16] where the mean Hb F levels were 8.8%, 9.5% and 9.1% respectively.

The relatively higher levels of Hb F in the current study may be a possible indicator that those with low HbF have more severe disease and die very young from complications of SCA even before the diagnosis is established. This way, only the survivors are seen in the clinic and are now classified as having high Hb F levels and possibly less severe disease. A prospective study of a birth cohort and neonatal diagnosis will be able to clarify this assertion.

The mean age at diagnosis in this study was 2.8 (SD 2.9) years which is much younger than that in the Dakar [15] study which was 9.8 years with only 56.7% of their patients diagnosed by 10 years while 83% of the children in this study were diagnosed by 5 years. The differences in age at diagnosis could be because this region in East Africa is a malaria endemic area and children with sickle cell are more likely to present with some of the complications of falciparum malaria such as severe malaria anaemia and may be tested and diagnosed early. There is also a relatively high prevalence of sickle cell disease and perhaps the index of suspicion is higher among the clinicians here.

Just about 70% of our study participants had at least one episode of severe pain requiring a visit to hospital in the previous year. This is less than the 90% observed in the Dakar study. One possibility is that the study included young children who may not be able to articulate the severity of their pain clearly, and so the caretakers may not bring them to hospital for pain. We found that the number of pain episodes was significantly related to the HbF levels which is similar to what Platt found that low HbF levels were significantly associated with number of pain episodes per year [20].

Complications such as cerebrovascular accidents (CVA), avascular necrosis and priapism were reported in only 10% of the study participants which is lower than what was found in Dakar (25%) [15] possibly because many of these occur in older patients. The Dakar study also had a smaller sample size of 60 patients and participants were prospectively followed up. Of key interest was that the Dakar study did not report any incident of CVA and yet this was the main complication in the present study. However, when our geographic location is put into consideration, this is where the Bantu haplotype is mainly found, and this has been associated with more severe disease.

Only 32% of the patients had never received a blood transfusion which is less than half of what was described in Dakar where 68.3% of their patients had never been transfused. The difference in HbF levels between those who had never been transfused and those who had received a transfusion was not statistically significant. One possibility why so many of the children have received transfusion may be the high malaria transmission in the country.

Although the relationship between high HbF as a categorical value and several markers of disease severity was not statistically significant, when the correlation between HbF levels as a continuous variable and the same markers of disease severity was examined, a significant relationship was clearly observed with the age at diagnosis of SCA, the number of transfusions, hospitalisations and the number of severe pain episodes in the past year. A mathematical modelling of the data may help in determining the minimum cut-off HbF level in our setting that may be associated with less severe disease. Knowledge of such a level will be important for designing trials of treatments that are used to raise HbF level.

This study had a number of limitations. Many of the markers of disease severity depended on the participants’ ability to remember events in the past. Thus, the ability to recall some of these clinical events and the unavailability of some medical records to ascertain the severity of symptoms may have affected our results. Any documentation would have been useful in defining the actual diagnosis for the previous admissions. Secondly, children who were severely anaemic at enrolment were excluded because the method of estimation of HbF level would be inaccurate when the Hb level is below 5 g/dl. This means some of the patients with severe disease may have been excluded. We also included all four patients who had been started on hydroxyurea; however, they had been on this therapy for a median of two weeks, and so the effect on HbF level would be minimal. Lastly, we did not examine for thalassemia and sickle cell haplotypes which may influence disease severity.

## Conclusions

One third of the patients with sickle cell anaemia in Mulago Hospital have high levels of HbF. High HbF is associated with less severe disease independent of age; including age at diagnosis, number of all cause hospitalisations and transfusions. We suggest that HbF levels should form part of the initial investigations in patients with sickle cell anaemia as this may have value in counselling on patient follow up, prognosis and also consideration for the preferential use of hydroxyurea. A mathematical modelling of the data from this study may help in determining the minimum cut-off HbF level in our setting associated with less severe disease. This level may be aimed at especially when considering treatments that are used to raise HbF level.

## Abbreviations

SCA: Sickle cell anaemia; SCC: Sickle cell clinic; HbF: Foetal haemoglobin.

## Competing interests

The authors declare that they have no competing interests.

## Authors’ contributions

LM, CMN, RI & HD contributed to the design of the study and assisted with data analysis. LM coordinated the study and supervised the enrolment of patients. All authors contributed to the interpretation of data, preparation of the manuscript and approved the final version.

## Authors’ information

LM: MBChB, M.Med (Paed)

CMN: MBChB, M.Med (Paed) is the head of the Sickle Cell Clinic in Mulago Hospital.

RI: MBChB, M.Med (Paed), PhD is a Paediatric neurologist in Mulago hospital and Clinical Research Paediatrician with the centre for Tropical Medicine, University of Oxford

HD: MBChB, M.Med (Int Med) is a haematologist in Mulago Hospital.

## Pre-publication history

The pre-publication history for this paper can be accessed here:

http://www.biomedcentral.com/1471-2326/12/11/prepub
